# Novel Strategies for the Treatment of Heart Failure

**DOI:** 10.5041/RMMJ.10078

**Published:** 2012-04-30

**Authors:** Izhak Kehat

**Affiliations:** The Clinical Research Institute at Rambam (CRIR), the Heart Institute, Rambam Medical Center, Haifa, Israel and the Department of Physiology, Bruce Rappaport Faculty of Medicine, Technion-Israel Institute of Technology, Haifa, Israel

**Keywords:** Calcium, excitation–contraction, heart failure

## Abstract

Heart failure is a leading cause of morbidity and mortality with a prevalence that is rising throughout the world. Currently the pharmaceutical therapy of heart failure is mainly based on inhibition of the neurohumoral pathways that are activated secondary to the deterioration of cardiac function, and diuretics to alleviate the salt and water overload. With our increasing understanding of the pathophysiology of heart failure, it is now clear that the macroscopic and functional changes in the failing heart result from remodeling at the cellular, interstitial, and molecular levels. Therefore, emerging therapies propose to intervene directly in the remodeling process at the cellular and the molecular levels. Here, several experimental strategies that aim to correct the abnormalities in receptor and post-receptor-function, calcium handling, excitation and contraction coupling, signaling, and changes in the extra-cellular matrix in the failing heart will be discussed. These novel approaches, aiming to reverse the remodeling process at multiple levels, may appear on the clinical arena in the coming years.

Heart failure is a leading cause of morbidity and mortality with a prevalence that is rising throughout the world.^1^ It is estimated, for example, that in Europe around 10 million people are suffering from this disease. Despite some progress in medical treatment within the last 10 years, morbidity and mortality of congestive heart failure are still high: 70%–80% of patients suffering from heart failure will die within the next 8 years.^2^ The reasons for the increase in incidence include the aging population and the increase in the cardiovascular risk factors obesity, diabetes, and hypertension. Paradoxically, the prevalence of heart failure remains high as more patients survive myocardial infarctions and fewer are dying from lethal arrhythmias.

After a myocardial infarction, the ventricle undergoes a progressive pathological and anatomical transformation resulting in a vicious cycle of left ventricular dilation, eccentric hypertrophy, and reduced function. Macroscopically these changes manifest as thinning of the infarct scar and, ultimately, an alteration of the left ventricular geometry to a spherical globe. These changes are collectively termed cardiac remodeling.^3^

Although the term cardiac remodeling was initially coined to describe the changes that transpire following myocardial infarction,^4^,^5^ it is clear that very similar processes are taking place following other types of injury such as occur with pressure overload (aortic valve stenosis, hypertension), volume overload (valvular regurgitation), inflammatory disease (myocarditis), and idiopathic dilated cardiomyopathy.^6^ Although the etiologies of these diseases are different, they share molecular, biochemical, and cellular processes to collectively change the shape and function of the myocardium. Therefore, therapies that target the remodeling process itself are important for all of these conditions.

Currently the pharmaceutical therapy of heart failure is based on inhibition of the neurohumoral pathways that are activated secondary to the deterioration of cardiac function, diuretics to alleviate the salt and water overload, and other strategies to mitigate predisposing, aggravating, or triggering factors. With our increasing understanding of the pathophysiology of heart failure, it is now clear that the changes in size, shape, and function of the heart that occur following injury result from remodeling at the cellular, interstitial, and molecular levels.^7^ Therefore, emerging therapies propose to intervene directly in the remodeling process at the cellular and the molecular levels.

Several pathophysiological phenomena characterize heart failure and appear to contribute to the progression of the disease. These include alterations in β-adrenergic receptor signaling due to desensitization, impaired calcium homeostasis, reduced excitation–contraction coupling, and altered energetics. Examples for future possible interventions in these processes that can ameliorate heart failure will be given here. This short review does not aim to discuss tried and tested approaches for the treatment of heart failure, nor can it give a comprehensive list of all possible approaches for heart failure. Rather, examples for future and emerging therapies targeting several pathophysiological pathways will be highlighted.

## BEYOND G-PROTEIN-COUPLED RECEPTOR (GPCR) BLOCKADE

Currently, the most effective treatments for heart failure are blockade of the β-adrenergic β1 receptors (β1AR) and angiotensin II type 1A receptors (AT1aR), which are both G-protein-coupled receptors (GPCRs). When stimulated by circulating catecholamine and angiotensin II, respectively, β- and AT1-receptors activate an associated G-protein (Gs for βARs and Gq for AT1aRs). This activation leads to stimulation of downstream signaling via generation of second messengers.^8^

Heart failure is characterized by long-term desensitization of the β-adrenoreceptors. The desensitization is mediated by phosphorylation of residues in the C-terminal tail of the activated receptor by a family of G-protein-coupled receptor kinases (GRKs). The phosphorylation of the receptors by GRKs enhances their affinity for proteins called β-arrestin. The signal is inhibited by blocking the interaction and uncoupling of the receptor and the corresponding G-protein, and by recruiting of enzymes that degrade second messenger molecules.^9^ In addition to their role in desensitization, β-arrestins are also important for internalization of the receptors.

Recent data also show that in addition to these uncoupling mechanisms, the recruitment of β-arrestin to βARs and AT1aRs also initiates a second wave of signaling independent of G-protein activation.^10^ Chronic Gs and Gq-protein signaling, occurring in failing hearts, is known to be harmful to the heart and contributes to heart failure. However it appears that β-arrestin-driven signaling by β-adrenergic receptors and angiotensin receptors may actually be cardioprotective, through transactivation of the epidermal growth factor receptor (EGFR).^11^ The development of ligands that activate a receptor to signal preferentially through one pathway, a process called biased agonism, may take advantage of this protective β-arrestin-mediated signaling. Indeed a clinically used β-blocker in heart failure, carvedilol, was shown to be a β-arrestin-biased ligand for β1-adrenoreceptors, which could explain its clinical advantages.^12^ Similarly a synthetically modified form of angiotensin II termed SII angiotensin was demonstrated to be an angiotensin type I receptor-biased agonist. It is unable to activate Gαq signaling but has the ability to recruit β-arrestin and activate signaling in a β-arrestin-dependent manner.^13^ Biased agonists for both the adrenergic and angiotensin receptor are being developed and may optimize therapy to maximize beneficial effects and minimize untoward effects. The potential therapeutic superiority of biased over unbiased ligands for the treatment of heart failure remains to be demonstrated in clinical studies.

**Figure 1:**
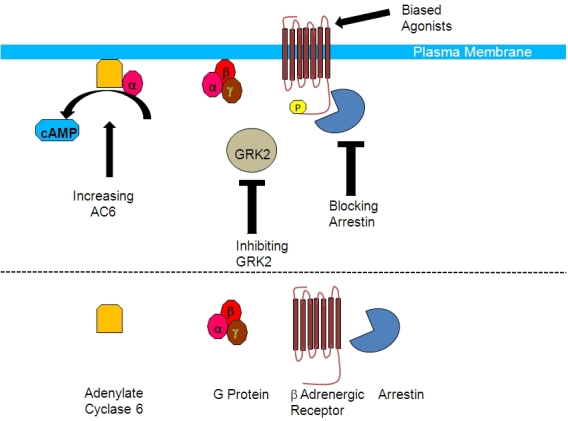
**Beyond G-protein-coupled receptor blockade.** Adrenergic β1 receptors signal through G-proteins (subunits αβγ) to activate adenylate cyclase 6. Desensitization of the receptors occurs through phosphorylation of its C-terminal loop by GRK2, which mediates binding of arrestins. Novel interventions include design of a biased agonist that specifically targets one second messenger over another, inhibition of GRK2 or inhibition of the arrestin–β receptor interaction, to prevent desensitization or activation of adenylate cyclase 6 to increase contractility.

The failing heart is characterized by alterations in β-adrenergic receptor signaling due, at least in part, to increased G-protein-coupled receptor kinase 2 (GRK2) activities. Initially, the up-regulation of GRK2 observed after cardiac injury is probably a protective mechanism intended to defend the heart from the noxious effects of excessive catecholamines, by reducing the signaling from the receptors. However, over time, the chronic receptor desensitization by GRK2 likely becomes maladaptive. Therefore, limiting βAR desensitization by GRK2 inhibition in heart failure may be therapeutic.^14^ One strategy for GRK inhibition may be to use a peptide derived from the carboxyl terminus of GRK2 known as the βARKct that can displace endogenous GRK2 from the membrane and prevent desensitization of the receptor.^15^ To assess this approach GRK2 inhibition was tested in rabbits in a study where adenovirus encoding for βARKct was administered into the coronaries at the time of myocardial infarction (MI). Three weeks post-gene transfer, GRK2 inhibition resulted in prevention of left ventricular (LV) adverse remodeling, improvement of cardiac contractility, and preservation of βAR signaling and function.^16^ Similarly, left ventricular remodeling was reversed by adeno-associated virus encoding for βARKct gene therapy in a pig model of heart failure.^17^ This and other studies make the βARKct a promising candidate for future application in human heart failure (Figure 1).

## “FIXING” CALCIUM HANDLING IN FAILING HEARTS

**Figure 2:**
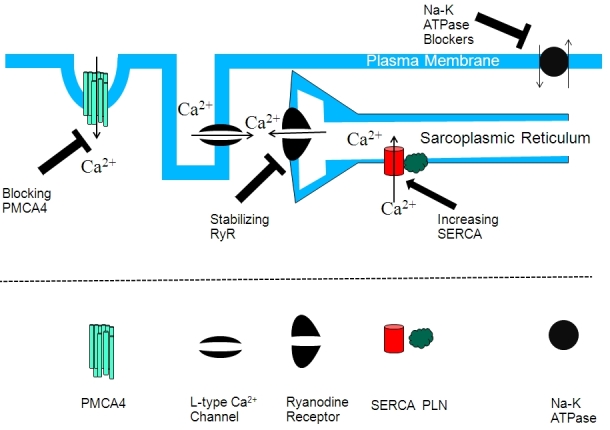
**Correcting calcium handling in failing hearts.** Opening of the L-type calcium channels allows calcium to enter the cytoplasm of the myocyte. This calcium elicits calcium release from the sarcoplasmic reticulum via the ryanodine receptors. Calcium is pumped back to the sarcoplasmic reticulum by the SERCA2 pump. The plasma membrane pump PMCA4 also allows calcium entry to the cell. Novel interventions aim at increasing the activity of SERCA2, preventing the leak of the ryanodine receptors, or blocking of PMCA4. Some drugs aim at both stimulation of SERCA and the inhibition of the Na^+^/K^+^-ATPase pump to increase intracellular sodium, which reduces the driving force for the sodium calcium exchanger.

Impaired calcium homeostasis is a prominent feature of the remodeling process and heart failure, and it manifests clinically as contractile dysfunction and development of arrhythmias.^18^ When compared to normal myocytes, the failing heart myocytes exhibit typical changes in intracellular calcium handling, including impaired extrusion of cytosolic calcium, reduced calcium loading in the cardiac sarcoplasmic reticulum (SR), and defects in the SR calcium release.^19^ These changes in calcium handling are thought to contribute to the impairment of cardiac contractile functions (Figure 2).^20^

Relaxation of the myofilaments after contraction is facilitated by two mechanisms of calcium extrusion: the rapid re-sequestration of calcium into the SR and calcium efflux outside of the cells through the plasma membranes. The sarco-endoplasmic reticulum calcium ATPase 2 pump (SERCA2) is localized on the SR membrane and is responsible for the re-uptake of calcium from the cytoplasm into the SR lumen. Since the amount of calcium released through the ryanodine receptors (RyR) during each cardiac cycle is proportional to the calcium content of the SR, the SERCA2 activity is a critical determinant of both relaxation (via calcium re-uptake into the SR) and contractility (via controlling the amount of calcium in the SR) in the cardiomyocytes.^21^ Indeed, experimental studies in animal models of heart failure have shown that increasing the expression of SERCA2a in cardiomyocytes normalizes intracellular calcium cycling, restores both relaxation and contractile function, and results in significant improvement in survival.^22^ Following the success of these animal studies, the Calcium Upregulation by Percutaneous Administration of Gene Therapy in Cardiac Disease (CUPID) trial enrolled 39 patients to receive intracoronary adeno-associated virus type 1 encoding for SERCA2 or placebo.^23^ The prevalence of neutralizing antibodies for adeno-associated virus serotype 1 in the population resulted in exclusion of about half of the patients screened for the trial. The patients were divided to control and to low-, medium-, and high-titer groups. Significant increases in time to clinical events and decreased frequency of cardiovascular events were observed at 12 months (hazard ratio = 0.12; *P* = 0.003), and the mean duration of cardiovascular hospitalizations over 12 months was substantially decreased in the high-dose treatment group versus placebo. According to the guidelines for treatment of patients with low ejection fraction, and due to concerns about arrhythmias, patients were implanted with defibrillators. There were no untoward safety findings, and no increase in arrhythmias was reported. Thus the CUPID study demonstrated safety and suggested benefit of adeno-associated virus type SERCA2 delivery in advanced heart failure. These promising preliminary results encourage larger trials to test clinical efficacy of this approach.

In the myocardium calcium is not only essential for contraction and relaxation but also has an important role as a second messenger in signal transduction pathways. This observation is somewhat counter-intuitive since the cardiomyocyte calcium concentration fluctuates from a resting diastolic level of 100 nM to a peak systolic level of 1 μM at every cycle. Variations in the frequency of the oscillations and spatial locations likely determine these “non-contraction–relaxation” related calcium signals. The local calcium signals are probably decoded by the effectors, usually calcium/ calmodulin-binding proteins, which translate the calcium signals to some specific actions.^24^ The calcium ATPases (also known as calcium pumps) are major participants in this process. These pumps are membrane-bound and therefore are responsible for transporting calcium ions across the membrane. In addition to the sarcoplasmic reticulum ATPase (SERCA) pump, cardiomyocytes possess a plasma membrane calcium ATPase (PMCA) pump. Isoform 4 of the PMCA (PMCA4), which is expressed in all cell types, is localized in the caveolae in cardiomyocytes,^24^ a compartment that contains a large number of signaling molecules. In this regard PMCA4 is uniquely situated to target the calcium signal, and it is hypothesized that PMCA4 is the calcium pump responsible for regulating calcium signaling in the heart and is not involved in excitation–contraction coupling. Support for this hypothesis came from the generation of cardiac-specific inducible PMCA4 transgenic mice that overexpress PMCA4 in cardiomyocytes.^25^ The hearts of these mice displayed normal global calcium transient and cellular contraction levels but a reduced cardiac hypertrophy following experimental pressure overload. Specific agents that can regulate the function of PMCA4 are being developed and may provide a novel therapeutic approach that aims at correcting the abnormal calcium-induced signaling in heart failure.

One of the calcium-sensing proteins in the heart is called S100A1, and it is a member of the EF-hand calcium-binding S100 protein family. As a calcium sensor protein it co-localizes and interacts with the SERCA2/phospholamban complex and modulates both systolic and diastolic ryanodine receptor function and cardiomyocyte SR calcium release, respectively.^26^ Failing hearts are characterized by progressively diminished S100A1 protein levels, and these low levels inversely correlate with the severity of the disease.^26^ These observations suggest that the down-regulation of S100 protein may be pathological. Indeed, S100A1 knock-out mice showed enhanced susceptibility to functional deterioration in response to chronic cardiac pressure overload stress and ischemic damage.^27^,^28^ In contrast, mice with overexpression of S100A1 are hypercontractile and maintained almost normal left ventricular function following myocardial infarction.^28^ Studies in a large-animal model of heart failure suggested that S100A1 may be an attractive target of cardiac gene therapy.^29^

The calcium leak through the ryanodine receptors is believed to contribute to the abnormal calcium cycling in failing hearts, and therefore this appears to be a target for treatment. In addition to reducing the sarcoplasmic reticulum calcium load, a leak may also trigger arrhythmias and increase energy consumption. A pharmacological agent, JTV519, can reduce the ryanodine receptor calcium leak, and this was shown to preserve contractile performance in a heart failure animal model.^30^ JTV519 was originally suggested to increase the binding of calstabin2 to RyR2. However, the original molecule JTV519 was not entirely specific to the ryanodine receptor and blocked in addition the L-type calcium channels and potassium channels. Another molecule S107 was shown to inhibit arrhythmias in a catecholaminergic polymorphic ventricular tachycardia mouse model.^31^ The effects of S44121, a more ryanodine leak-specific agent, is currently being analyzed in patients with congestive heart failure who are at risk for ventricular arrhythmias in a phase 2 clinical study.

## TARGETING CONTRACTILITY IN HEART FAILURE

The β-adrenoreceptor transduces the signal through Gs protein to adenylate cyclase, which leads to increased generation of cyclic adenosine monophosphate (cAMP), which then interacts with protein kinase A (PKA) and other intracellular effector proteins. Currently, 10 different isoforms of adenylate cyclase have been cloned and characterized in mammals, of which the adult human left ventricle appears to express predominantly adenylate cyclase isoform 6 (AC6). Failing human hearts have reduced amounts of basal cAMP and impaired cAMP generation in response to agonist stimulation.^32^ However, results of clinical trials that aimed to increase β-adrenoreceptor activation by the agonist dobutamine or to increase the cAMP content through inhibition of the phosphodiesterase that breaks it down by milrinone have been disappointing. A possible explanation for the failure of these approaches may be that both these agents would be predicted to increase the intracellular levels of cAMP, which may provoke lethal cardiac arrhythmias. Along with these observations, mice that were engineered to overexpress the β-adrenoreceptor or Gα protein displayed initial sustained increases in heart rate and ventricular contractile function, followed by ventricular dilation, myocardial fibrosis, and heart failure.^33^ In contrast, there were distinct differences in mice with cardiac-directed expression of AC6—despite 20-fold excess cardiac AC6 protein, there was no increase in heart rate or left ventricular function in unstimulated animals. Moreover, the animals displayed improved responsiveness to β-adrenoreceptor stimulation by showing marked increases in heart rate and contractile function. Most importantly, unlike mice with cardiac-directed β-adrenoreceptor or Gα, there was no decline in function or abnormalities of cardiac structure or histology even in old mice. The precise mechanisms for these striking differences in effect were not determined. Exogenous gene transfer will be required if AC6 is ever to be applied in the treatment of clinical heart failure, and so far clinical trials are lacking.

Istaroxime is a non-cardiac glycoside that has inhibitory effects on the Na^+^/K^+^-ATPase pump, and it is suggested to possess SERCA-stimulatory abilities.^34^ The inhibition of the Na^+^/K^+^-ATPase pump increases intracellular sodium, which reduces the driving force for the sodium calcium exchanger (NCX) and decreases calcium extrusion from the cell. The increased sodium may actually stimulate the NCX to function in the reverse mode and transport calcium into the cell in exchange for sodium. The calcium influx into the cytosol is expected to increase contractility, but may also be harmful in the failing heart which already has elevated diastolic calcium levels. This mechanism likely explains the modest benefit of drugs such as digoxin in heart failure. Therefore the additional capability to promote SERCA activity and the uptake of calcium to the sarcoplasmic reticulum may be crucial to the success of an inotropic agent that blocks the Na^+^/K^+^-ATPase pump. In several animal studies, istaroxime increased the maximum rates of rise and fall in left ventricular pressure and decreased end-diastolic pressure and volume without a change in heart rate and blood pressure. Most importantly, these inotropic and lusitropic (relaxation) effects were different from those of digoxin and have not been associated with an increase in myocardial oxygen consumption.^35^ In the HORIZON trial (a randomized, double-blind, placebo-controlled study that recruited 120 patients with relatively mild heart failure that did not require inotropes), an intravenous infusion of istaroxime resulted in an increase in systolic blood pressure and a transient increase in cardiac index, without a change in ejection fraction.^36^ These hemodynamic effects reflect the predicted inotropic and lusitropic properties of the drug; however, much larger trials are of course needed to demonstrate clinical efficacy.

A novel potential signaling target for excitation–contraction coupling may be protein kinase C (PKC). PKC was reported to phosphorylate the L-type calcium channel, phospholamban (PLN), and possibly the ryanodine receptor (RyR) as well.^37^ However, the exact physiological significance of PKC phosphorylation of these calcium-handling regulators remains unknown. In the mouse heart activation of PKCα suppresses sarcoplasmic reticulum calcium cycling by phosphorylating protein phosphatase inhibitor 1. Hearts of PKCα-deficient mice are hypercontractile, whereas those of transgenic mice overexpressing PKCα are hypocontractile.^38^ A study showed that phosphorylated phosphatase inhibitor 1 dissociated from protein phosphatase-1 and -2A and the resulting enhanced protein dephosphorylation activity lowered the phosphorylation level of PLN. Similarly short-term pharmacological inhibition of the conventional PKC isoforms significantly augmented cardiac contractility in wild-type mice and in different models of heart failure *in vivo*, but not in PKCα-deficient mice.^39^ Thus, PKCα functions as a nodal integrator of cardiac contractility by sensing intracellular calcium and signal transduction events, which can modify contractility. PKCα inhibitors are available and have shown benefit in animal models. Further studies are needed in order to assess the potential use of a PKC inhibitor in the failing heart.

A different approach to improve excitation–contraction coupling would be to improve force generation without altering the calcium transient in the myocyte. Stimulation of the myosin ATPase is expected to accelerate the release of the weak actin–myosin cross-bridge and promotes transition to the force-producing state of the cross-bridge.^35^ As more cross-bridges are activated the contractile force increases. Indeed several such myosin ATPase-stimulatory agents were demonstrated to increase the fractional shortening of myocytes without increasing the intracellular calcium transients. In initial studies in dog models of heart failure, one such molecule, omecamtiv mecarbil, increased stroke volume and cardiac output and decreased LV end-diastolic pressure and heart rate without increasing myocardial oxygen demand.^40^ Omecamtiv mecarbil binds to the myosin catalytic domain and operates by an allosteric mechanism to increase the transition rate of myosin into the strongly actin-bound force-generating state and accelerates actin-dependent phosphate release, which is the rate-limiting step in the actin–myosin ATPase cycle in cardiomyocytes.^41^ In small clinical studies omecamtiv mecarbil infusion resulted in dose- and concentration-dependent increases in stroke volume, fractional shortening, and ejection fraction.^35^ Omecamtiv mecarbil also increases the left ventricular systolic ejection time, and there is some concern that this increase may hamper the diastolic filling, although no untoward effects have been demonstrated so far. In addition, signs and symptoms of myocardial ischemia appeared with high-dose administration of the drug. This raises concern for potential ischemia during omecamtiv mecarbil therapy, especially in patients with coronary artery disease and at high heart rates.^42^

## TARGETING MYOCARDIAL SUBSTRATE METABOLISM IN HEART FAILURE

Alterations in the energetic balance and substrate utilization have an important role in heart failure, and a shift from fatty acid to glucose as the preferred substrate and a decline in the levels of ATP accompany the transition to failure. These changes are probably not due to changes in the substrate availability, as the coronary circulation provides an excess of substrates, but rather result from changes in substrate flux and modification of the enzymatic repertoire in the cells. These changes are further exacerbated by the increasing metabolic demands in the failing heart. As heart failure progresses, the compensatory hyperadrenergic state leads to an elevation of plasma free fatty acid levels. This elevation impairs the normal adaptive metabolic response and leads to up-regulation of free fatty acid metabolism and increased oxygen consumption, thus creating a vicious cycle with further myocardial deterioration. Carnitine palmitoyltransferase-1 (CPT1) is a key enzyme regulating the uptake of fatty-acyl-CoA, the activated form of free fatty acid, into the mitochondria.^43^ Therefore, a reduction in the activity of this enzyme results in a shift in substrate usage from free fatty acid to glucose in the myocardium. Etomoxir is an irreversible inhibitor of mitochondrial CPT1 and long chain free fatty acid oxidation. Blockade of CPT1 results in a decline in the intracellular levels of acetyl-CoA, relieves the inhibitory effect on glycolysis, and results in increased activity of pyruvate dehydrogenase and phosphofructokinase, and enhanced glycolysis and glucose oxidation.^43^ A clinical trial using etomoxir was stopped prematurely because the use of this agent was associated with elevation in liver function tests; however, a small study with another CPT inhibitor, perhexiline, showed benefit in ejection function and myocardial energetics.^44^

AMP-activated protein kinase (AMPK) is an AMP-sensitive enzyme which is expressed in many tissues, including the heart. AMPK is a key regulator of the metabolic pathways, and it ultimately modifies ATP-consuming pathways. AMPK inhibits CoA carboxylase, reduces the production of malonyl-CoA, and thus increases CPT1-dependent fatty acid oxidation to increase energy production. AMPK also stimulates glucose uptake by stimulating the translocation of GLUT4 transporters. The activation of AMPK is therefore a response to low energy states such as ischemia and exercise. Currently, the only AMPK-modulating drugs act indirectly. The anti-diabetes drug metformin has been associated with reduced cardiovascular death and myocardial infarction in diabetics and is known to activate AMPK.

## ALTERING THE CHANGES IN THE EXTRA-CELLULAR MATRIX

The myocardial extra-cellular matrix (ECM) is a complex microenvironment containing a large number of matrix proteins, signaling molecules, proteases, and different cell types that play a fundamental role in the myocardial remodeling process. The remodeling process in the failing heart is commonly referred to as fibrosis and is histologically apparent as an increase in fibrillar collagen and myofibroblast proliferation in the heart. The dynamic changes occurring within the interstitium can directly contribute to the adverse myocardial remodeling following MI, with hypertensive heart disease and with intrinsic myocardial disease such as cardiomyopathy.^45^

Data from trials of standard therapy of heart failure support the notion that myocardial fibrosis can be targeted with beneficial clinical results. For example, data from the RALES and EPHESUS trials showed that the use of mineralocorticoid receptor antagonists in heart failure patients resulted in reduced fibrosis, less remodeling, and better clinical outcomes. The synthesis of collagen in the heart is regulated by myofibroblasts. The origin of these cells is still unclear, but they may result from growth factor-induced differentiation of resident fibroblasts or recruitment of cells to the heart.^46^ Several studies have suggested that TGF-β induces the trans-differentiation of fibroblasts to myofibroblasts. Therefore, drugs that inhibit the TGF-β receptor or pathway may be useful to interfere with the fibrotic process. For example, in an experimental rat model of myocardial infarction, treatment with a TGF-β type I receptor inhibitor led to attenuation of myocardial remodeling and LV dysfunction.^47^

## CONCLUSIONS

Heart failure results from alterations that are not necessarily adaptive to the initial insult, but pathologic and potentially self-perpetuating in a progressive vicious circle. These alterations include, but are not limited to, changes in receptor and post-receptor function, calcium handling, excitation and contraction coupling, signaling, and changes in the extra-cellular matrix. Novel approaches to target these pathways at multiple levels are emerging and may appear on the clinical arena in the coming years.

## Abbreviations:

AC: adenylate cyclase;
AMPK: AMP-activated protein kinase;
AT1aR: angiotensin II type 1A receptor;
β1AR: β1 adrenergic receptor;
cAMP: cyclic adenosine monophosphate;
CPT1: carnitine palmitoyltransferase-1;
ECM: extra-cellular matrix;
EGFR: epidermal growth factor receptor;
GPCR: G-protein-coupled receptor;
GRK: G-protein-coupled receptor kinase;
NCX: sodium calcium exchanger;
PKC: protein kinase C;
PMCA: plasma membrane calcium ATPase;
RyR: ryanodine receptor;
SERCA2: sarco-endoplasmic reticulum calcium ATPase 2 pump;
SR: sarcoplasmic reticulum.
